# Effect of Calcium Hydroxide and Nixtamalization Time on the *In Vitro* Starch and Protein Digestibility of Traditional Maize Tortillas

**DOI:** 10.1007/s11130-024-01245-z

**Published:** 2025-02-10

**Authors:** Izxayana Escamilla-Urbina, Alfonso Totosaus-Sánchez, M. Eva Rodríguez-Huezo, E. Jaime Vernon-Carter, Jose Alvarez-Ramirez

**Affiliations:** 1 Departamento de Ingeniería Química y Bioquímica, TecNM/TES Ecatepec, Estado de México, Av. Tecnológico S/N, Col. Valle de Anáhuac, Ecatepec de Morelos, C.P. 55210 México; 2 Departamento de Ingeniería de Procesos e Hidráulica, Universidad Autónoma Metropolitana- Iztapalapa, Apartado Postal 55-534, Iztapalapa, 09340 CDMX México

**Keywords:** Maize tortilla, Multivariate analysis, Nixtamalization conditions, Digestibility

## Abstract

This work addressed the traditional nixtamalization processing conditions on the *in vitro* protein and starch digestibility of tortillas. Two levels of lime addition (1 and 2 g/100 g db) and three different cooking times (30, 45 and 60 min) were considered. FTIR analysis was used to evaluate the organization of water, proteins and starch in the tortilla matrix. Lime concentration impacted the random secondary structure formation of the protein, and cooking time the starch ordered structures content. Higher lime concentration led to lower protein digestibility, but longer cooking time increased it. Highest protein hydrolysis (74.36 ± 3.72%) was achieved in T_1,60_ after 120 min of enzymatic digestion. Higher RDS contents were found as lime concentration increased, probably due to starch chain fragmentation at all cooking temperatures. A multivariate analysis indicated that both the lime concentration and cooking time had a major effect on the tortilla’s digestibility. Both protein and starch digestibility can be modulated by using appropriate lime concentrations and cooking temperatures.

**Supplementary Information:**

The online version contains supplementary material available at 10.1007/s11130-024-01245-z.

## Introduction

Nixtamalization is a multi-stage process that consists of the following steps: cooking the dry maize grains in a calcium hydroxide solution, steeping, draining and water rinsing of the grains for removing the grain pericarp, any remaining organic components and excess alkali, milling of the grains to produce a soft malleable fresh dough called masa, and hand-pressing or machine-molding the dough, which is baked to obtain the tortillas. Traditional nixtamalization may be carried out at an artisanal level, which usually ends with the baking of the dough into tortillas, or in large industrial plants, where the fresh masa is dried and ground to obtain a nixtamalized flour for producing commercial tortillas. Artisanal tortillas have been reported as having higher nutrients and bioactive compounds contents than tortillas produced by large food retailers using nixtamalized flour [1]. Industrial level nixtamalization is generally carried out under harsher conditions (higher lime concentrations and longer cooking times) for shortening processing times than at artisanal level.

It is estimated than in Mexico there are about 100,000 small traditional nixtamal artisanal mills supply ~ 70% of the tortillas. The prevalence of overweight in adults is ~ 39.1% and of obesity ~ 36.1% [2], while overweight in children overweight is of ~ 22.2% (age 0–4 years) and of ~ 35.6% (age 5–11 years). It is agreed that there is a direct link between intake of different starches and sugars with body weight management and metabolic disturbances. The hydrothermal treatments of starch destroy its granular structure, which may lead to rapid digestion and absorption in the small intestine [3]. There are indications of potential detrimental effects caused by high intakes of rapidly digestible starch (RDS), but also potential beneficial effects ingesting slowly digestible (SDS) and resistant (RS) starches [4]. Recent studies indicate that SDS reduces post-prandial response [5] and that RS reduces the risk of diet-related disorders such as obesity and diabetes is promising, but these findings are still inconclusive [6].

Given the lack of studies dealing with the impact of traditional nixtamalization processing conditions on starch and protein digestibility, the aim of this work was to address the effect of lime concentration and cooking times on the *in vitro* protein and starch digestibility of maize tortilla. To this end, FTIR analysis used in combination with *in vitro* digestibility assays, were used in order to gain insights regarding the mechanisms underlying the digestibility properties caused by the nixtamalization conditions on the relative amount the starch fractions (RDS, SDS and RS) generated [7], and on starch and protein digestibility [8].

## Materials and Methods

The Materials and methods section is presented as supplementary material.

## Results and Discussion

### FTIR Analysis

The FTIR spectrum of the T_x,y_ is shown in figures S1.a and S1.b in Supplementary Material. The broadband 3700 –3000 cm^−1^ is ascribed to the interactions of the water molecules with the tortilla components. The hydroxyl group OH interacts with biopolymers, leading to a structuration of water in regular configurations. Two small peaks at about 2950 and 2850 cm^−1^ correspond to aliphatic groups, which are linked to the presence of lipids in the tortilla structure. The FTIR band 1700 –1600 cm^−1^ corresponds to the Amide I linked to the stretching of the C = O group of proteins. The Amide I band is commonly considered to gain insights into the secondary structure of proteins. The region with a peak at about 1020 cm^−1^ is considered a fingerprint band for starch and provides information on the short-order structure of starch chains [9].

The OH band is composed of three main contributions at 3220, 3393 and 3540 cm^−1^ (see Figure S2 in Supplementary Material). The relative contribution of these bands is presented in Table 1. The shift to higher wavenumbers indicates the decrease of the bonding strength of water molecules with the polymeric matrix [10]. The band at 3220 cm^−1^ was linked to fully bonded water of low density with a coordination number close to four like ice. The band at 3400 cm^−1^ denotes water with an average degree of connection greater than for dimers and trimers, but lower than for ice (clusters). The peak at about 3540 cm^−1^ was linked to water molecules with poor connection to their environment and can be seen as liquid-like structures. The lime concentration and the cooking time affected mainly the contribution of the bands at 3220 and 3540 cm^−1^. The cooking time led to an increase of the low-density bonded water (3220 cm^−1^) and to an increase of the liquid-like water structures (3540 cm^−1^). This effect could be linked to an increase of the starch gelatinization with the lime concentration and the cooking time [11]. Starch hydrogels are amorphous structures with a high capacity of retaining liquid water in a complex starch chain network. The numerical deconvolution of the Amide I region revealed the contribution of two and three protein secondary structures for the low and high lime additions (Table 1). Coils and β-sheets are the contributions for the lime concentration of 1 g/100 g db. The content of the coils decreased with the cooking time, whilst the content of the β-sheets increased. The increase of the β-sheets content can be ascribed to the binding of calcium ions, forming a plated structure mediated by a cross-linking effect. In this way, the calcium ions would provide a long-range interaction to aggregate and stabilize the protein structure [12]. Lime addition at 2.0 g/100 g db induced the formation of random structures, due maybe to the hydrolysis of the protein moieties. It should be highlighted that random fractions are not a true secondary structure, but is the class of conformations that indicate an absence of regular secondary structure. In contrast to the lime addition at 1.0 g/100 g db, longer cooking time induced an increase of the coil, but left practically unaltered the content of β-sheets structures.

**Table 1:** FTIR analysis of the different tortilla formulations

Sample	3220 cm^−1^ (%)	3393 cm^−1^ (%)	3540 cm^−1^ (%)	Coils (%)	Random (%)	β-sheets (%)	995/1022	1044/1022
**T**_**1,30**_	46.39 ± 0.18^a^	43.32 ± 0.18	8.93 ± 2.69^c^	68.11 ± 0.21^b^	0.04 ± 0.17^b^	31.83 ± 1.32^c^	0.37 ± 2.02^c^	0.19 ± 2.02^c^
**T**_**1,45**_	42.88 ± 0.18^b^	44.63 ± 0.23	10.24 ± 3.75^b^	66.89 ± 0.36^a^	0.00 ± 0.23ª	33.10 ± 1.46ª	0.27 ± 3.11ª	0.17 ± 2.02^c^
**T**_**1,60**_	40.72 ± 0.18^c^	44.63 ± 0.02	12.82 ± 3.72ª	60.76 ± 0.41^a^	0.00 ± 0.25^a^	39.23 ± 1.67^b^	0.24 ± 3.16^b^	0.20 ± 2.02^c^
**T**_**2,30**_	43.22 ± 0.18^a^	44.88 ± 0.19^c^	10.48 ± 3.13^c^	39.47 ± 0.65^b^	34.13 ± 0.18^a^	26.39 ± 1.86^a^	0.51 ± 3.75^a^	0.27 ± 2.02^c^
**T**_**2,45**_	41.64 ± 0.18^c^	45.75 ± 0.21^a^	10.90 ± 3.96^b^	40.57 ± 1.02ª	30.57 ± 0.15^b^	28.84 ± 2.65^b^	0.45 ± 3.85^b^	0.24 ± 2.02^c^
**T**_**2,60**_	42.25 ± 0.18^b^	45.25 ± 0.02^b^	10.89 ± 4.16ª	49.12 ± 0.96^b^	24.21 ± 0.21ª	26.65 ± 2.33^b^	0.71 ± 3.26ª	0.45 ± 2.02^c^

Values are means ± standard error, of three replicates. Superscripts with different letters in same column indicate significant differences (*P* ≤ 0.05). T_x,y_: tortilla with “x” content (g/100 g) of calcium hydroxide and “y” nixtamalization time

The FTIR ratio 995/1022 is indicative of hydrated starch structures and increased with both the lime addition and the cooking time (Table 1). Nixtamalization induces breakage and gelatinization of starch chains, resulting in a higher capacity of retaining water molecules [11]. The effect is more pronounced for the lime addition of 2.0 g/100 g db, suggesting that the calcium hydroxyl provokes the formation of partially gelatinized starch structures in the maize grain. On the other hand, the ratio 1044/1022 was scarcely affected by the cooking time for 1 g/100 g db of lime addition. The ratio was higher and increased with the cooking time for the lime addition of 2 g/100 g db. This suggests that the calcium ions are linked to the starch chains via crosslinking mechanisms to form ordered starch structures [13].

## *In Vitro* Digestibility

The kinetics of the protein hydrolysis for the T_x, y_ is displayed in figures S3.a and S3.b for lime additions of 1.0 and 2.0 g/100 g db. A first or zero order kinetics model was used to fit the least-square hydrolysis kinetics data. The model discrimination was made based on maximizing the F-value. In figures S3.a and S3.b, the best fitting exhibits zero-order kinetics for the 30 and 45 min of cooking time, and a first-order kinetics for the 60 min cooking time. Table 2 presents the estimated rate constants. Zero order reactions do not depend on the concentration, suggesting that the protein hydrolysis is controlled by diffusion. The increase of the cooking time to 60 min shifted the hydrolysis kinetics to first-order mechanisms where the hydrolysis rate depends on the protein availability. The results in Table 1 indicate that the cooking time modifies the protein structure via the formation of hydrolysates and disrupts the protein attachment to the starch granule. The combination of these effects would lead to easy access to the protein binding sites by the proteolytic enzymes. Table 2 presents the protein hydrolysis achieved after 120 min, indicating a marked increase with the cooking time. However, the effect was more pronounced for the low lime addition where the hydrolysis was about 74.36% for 60 min cooking time, in contraposition to 61.91% for the high lime addition. On the other hand, the protein hydrolysis was lower for the high lime addition. This reduction could be attributed to the formation of calcium-mediated cross-linked structures, which in turn limit the action of the proteolytic enzymes [14]. Overall, the results in Table 2 showed that the cooking time has a positive effect in the digestibility of proteins, while the lime concentration has a negative effect.

Figure S4 displays starch digestograms for the T_x,y_. It is apparent that the starch hydrolysis exhibits a piecewise zero-order kinetics pattern. This suggests that the hydrolysis of the starch contained in the tortilla structure is independent of the concentration, although dependent on the hydrolysis time. The starch-binding module and the surface-binding site of *α*-amylase facilitate the binding of the substrate to the active sites of the amylolytic enzymes, enhancing the starch hydrolysis. Barorouh et al. [15] reported that such sites are a noncatalytic module, which could interact with insoluble starch fractions. In this way, the zero-order kinetics can reflect limited access of enzyme to active sites in the starch granules [16]. High hydrolysis rates were displayed for digestion times smaller than about 40 min. The hydrolysis rate exhibited a marked decline for larger digestion times. This kinetics pattern indicates the presence of two digestible starch structures linked to rapid and slow hydrolysis rates. The first structure would correspond to gelatinized starch, which is easily accessible by amylolytic enzymes. The second structure would be given by granular structures, which are slowly hydrolyzed due to limitations in the starch-enzyme interactions [16].

**Table 2:** Parameters of *in vitro* digestibility of protein contained in the different tortilla formulations

Sample	Protein Kinetics Order	$k_p\times\:10^{-1}$ (see footnote)	Protein Hydrolysis (% at 120 min)
**T**_**1,30**_	0th	4.32 ± 0.18	51.53 ± 2.69^c^
		F = 458.23	
**T**_**1,45**_	0th	5.93 ± 0.23	68.56 ± 3.75^b^
		F = 566.30	
**T**_**1,60**_	1st	0.21 ± 0.02	74.36 ± 3.72ª
		F = 419.28	
**T**_**2,30**_	0th	4.26 ± 0.19	50.20 ± 3.13^c^
		F = 644.39	
**T**_**2,45**_	0th	4.75 ± 0.21	58.67 ± 3.96^b^
		F = 385.18	
**T**_**2,60**_	1st	0.26 ± 0.02	61.91 ± 4.16ª
		F = 187.99	

Values are means ± standard error, of three replicates. Superscripts with different letters in same column indicate significant differences (*P* ≤ 0.05). T_x,y_ : tortilla with “x” content (g/100 g) of calcium hydroxide and “y” minutes of boiling. $k_p$: Protein hydrolysis rate constant, with units %.min^−1^ for 0th -order kinetics and min^−1^ for 1st -order kinetics

The compact packing of starch chains can limit the access of the starch chains to the active sites of the enzyme, resulting in a decreased rate of starch fractionation [15]. In this way, the RDS fraction reflects the hydrolysis of starch structures that are easily accessible to amylolytic enzymes. Table 3 shows that the variations of the total starch were statistically non-significant (*p* < 0.05) for lime concentration of 1%. In contrast, the total starch showed a slight decrease for the lime concentration of 2%, an effect that could be caused by starch loss in the nixtamalization process and linked to an alkaline-mediated fragmentation of starch chains [12]. Table 3 also presents the estimated zero-order hydrolysis rates. The hydrolysis advance was higher for the high lime addition, which can be linked to the disruption of the tortilla structure by calcium ions, and hence to a facilitated access to starch binding sites by amylolytic enzymes. It has been postulated that the disruption of insoluble remnants (ghosts) by alkali treatment and the formation of calcium-mediated cross-linking structures play an important role in the *in vitro* digestibility and microstructural changes of maize starch [12]. The cooking time has an interesting effect as the transit from 30 to 45 min of cooking time increased the starch hydrolysis for both lime additions. However, the further increase of the cooking time to 60 min led to a regression of the of the hydrolysis advance. The cooking disrupted the structure of the maize grains, allowing an easy action of the amylolytic enzymes. At the same time, cooking leads to starch gelatinization and calcium-mediated cross-linking, which in turn hampers the access of the enzymes to the binding sites of the starch chains. Table 3 displays the RDS and SDS fractions, showing that the lime addition increased the RDS fraction, with a more visible effect for the highest lime concentration. It has been suggested that the increased RDS fraction can be ascribed to the fragmentation of the starch chains caused by the calcium hydroxide [12].

Our reported RDS values are lower (ranging from 12.19 to 46.12%) compared to the average value of 74.23% reported for five landraces studied by Acosta-Estrada et al. [17]. Such discrepancy can be attributed to the landrace that we used and to differences in processing conditions. Acosta-Estrada et al. [17] used 3 kg grain, 30 g lime, 9 L water, 95 °C with stirring (20 rpm), without indicating the cooking time, followed by 15 steeping. In our case we used 1 kg grain, 10–20 g lime, 2 L water, without stirring, using cooking times of 15, 30 and 60 min, followed by steeping for 12 h at room temperature. Artisanal nixtamalization of maize grains involves the grinding of the cooked grains after discarding the cooking liquor. The grinding is done with stone mills which produce coarsely ground maize grain fragments, which are sieved, and a fresh masa is obtained from which tortillas are made. The structure of the starch granules is not completely destroyed.

**Table 3:** Parameters of starch *in vitro* digestibility of the different tortilla formulations

Sample	$\:{\varvec{k}}_{\varvec{S},\varvec{f}\varvec{a}\varvec{s}\varvec{t}}\times\:{10}^{-1}$ (%.min^−1^)	$\:{\varvec{k}}_{\varvec{S},\varvec{s}\varvec{l}\varvec{o}\varvec{w}}\times\:{10}^{-1}$ (%.min^−1^)	TS (g.100 g^−1^ db)	RDS (%)	SDS (%)
**T**_**1,30**_	3.81 ± 0.21^b^	1.88 ± 0.17b	67.26 ± 1.45^a^	12.19 ± 1.32^c^	29.33 ± 2.02^c^
	F = 198.94	F = 26.77			
**T**_**1,45**_	7.35 ± 0.36^a^	2.88 ± 0.23ª	68.08 ± 1.63ª	20.15 ± 1.46ª	41.70 ± 3.11ª
	F = 475.74	F = 344.58			
**T**_**1,60**_	7.22 ± 0.41^a^	2.65 ± 0.25^a^	67.45 ± 1.37ª	17.92 ± 1.67^b^	36.89 ± 3.16^b^
	F = 691.29	F = 629.86			
**T**_**2,30**_	10.06 ± 0.65^b^	2.04 ± 0.18^a^	66.02 ± 1.16^a^	24.51 ± 1.86^a^	35.81 ± 3.75^a^
	F = 976.26	F = 206.28			
**T**_**2,45**_	16.20 ± 1.02ª	1.63 ± 0.15^b^	65.15 ± 1.05^ab^	46.12 ± 2.65^b^	31.42 ± 3.85^b^
	F = 196.23	F = 341.23			
**T**_**2,60**_	10.35 ± 0.96^b^	1.95 ± 0.21ª	64.16 ± 1.33^b^	31.82 ± 2.33^b^	34.16 ± 3.26ª
	F = 416.95	F = 145.31			

Values are means ± standard error, of three replicates. Superscripts with different letters in same column indicate significant differences (*P* ≤ 0.05). T_x, y_ : tortilla with “x” content (g/100 g) of calcium hydroxide and “y” minutes of boiling.$\:{k}_{S,fast}$: starch fast hydrolysis rate constant.$\:{k}_{S,slow}$: starch slow hydrolysis rate constant. RDS: Rapidly digestible starch. SDS: Slowly digestible starch

In industrial nixtamalization, the fresh masa is dried, milled and sieved for obtaining a nixtamalized flour, from which a masa and tortillas are obtained. The additional processing causes further destruction of the starch granular structure and produces a smaller particle size, destroying the starch granules to a greater extent. Dhital et al. [18] reported that the *in vitro* digestibility of maize starch kept an inversely square relation with granule size, leading to lower RDS contents in artisanal than in commercial tortillas. On the other hand, Sáyago-Ayerdi et al. [19] reported that artisanal freshly prepared tortillas exhibited a hydrolysis degree (%) ranging between 5.85 and 38.86% as hydrolysis time elapsed from 30 to 180 min, using a chewing/dialysis digestion system. These results are in line with the starch hydrolysis-time plot presented in Figure S.4 presented in the Supplementary Material for the 1.0% lime nixtamalization condition.

## Principal Component Analysis (PCA)

**Fig. 1:**
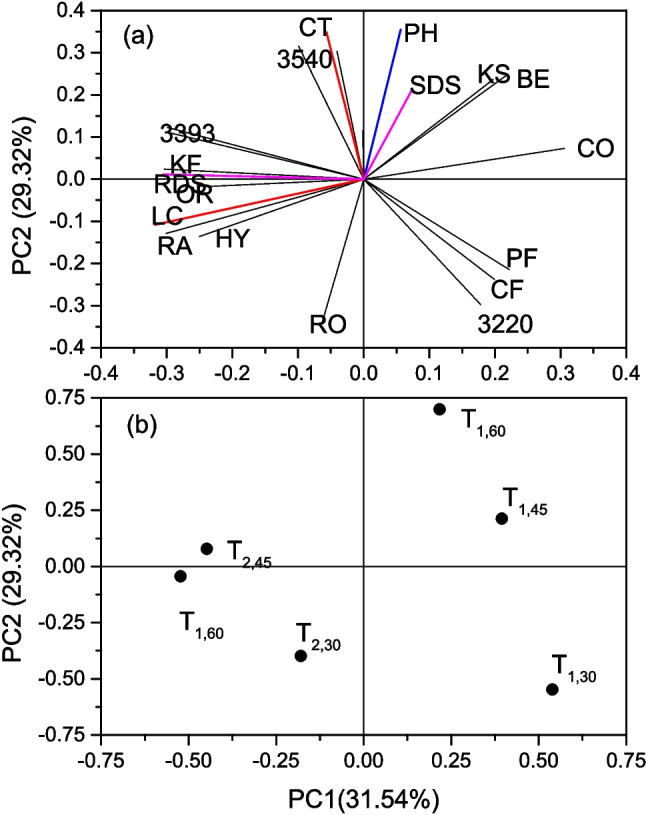
Principal Component Analysis (PCA) for the diifferent tortilla formulations (Tx,y). (**a**) Score plot of the 13 response variables defined in The Principal Component Analysis subsection. (**b**) Score plot of Tx,y, where x is the lime concentration and y is the cooking time

PCA was carried out to estimate the correlation between the six T_x,y_ formulations and the thirteen evaluated variables: LC: Lime concentration; CT: Cooking time; 3220, 3393 and 3540: Deconvolution components from the OH FTIR band. CO: Coils structures; RA: Random secondary structures; BE: $\:\beta\:$-sheet structures; KF: $\:{k}_{S,fast}$; KS: $\:{k}_{S,slow}$; RDS: Rapidly digestible starch; SDS: Slowly digestible starch; PD: Protein digestibility. The first two principal components accounted for the 31.51 and 29.32% of the total variability. Figure 1.a displays the distribution of the variables on the first two principal component plane. The red lines correspond to the control variables given by lime concentration (LC) and the cooking time (CT). The arrangement of these variables in the PC1/PC2 plane is nearly orthogonal, meaning that the effects of the lime concentration and the cooking time are mutually independent. The blue line corresponds to the contribution of protein digestibility (PD). This variable showed a strong alignment with the cooking time. The magenta lines correspond to the starch digestibility. The RDS is aligned with lime concentration, suggesting that increasing the added calcium hydroxide leads to an increase of starch that is rapidly digestible. This effect can be linked to a fragmentation of the starch by action of the hydroxide group, resulting in short starch chains that are more accessible to amylolytic enzymes. In contrast, the SDS line is closer to the cooking time, which indicates that an extended cooking of the nixtamalized maize grains induces a calcium-mediated crosslinking of the starch chains, and hence to obstruction for the enzymatic actions [17]. Other interesting alignment of variables are the following. The 3540 cm^−1^ is positively aligned with the cooking time, indicating that cooking promotes the formation of liquid-like water structures in the tortilla matrix. The formation of starch hydrated and ordered structures are positively aligned with the lime concentration, suggesting that calcium ions play an important role in the structuration of the starch chains.

## Conclusions

Lime concentration and cooking time are the two major variables that can be regulated to obtain tortillas with prescribed digestibility characteristics of starch and proteins. The results in this work showed that the nixtamalization conditions are major determinants of the *in vitro* digestibility characteristics of the low-cost traditional maize tortilla. Following the trend of population specific food products, traditional tortilla with tailored nutritional characteristics can be produced by a tuning of nixtamalization conditions. In this regard, the consumer’s acceptability should be evaluated to assess the limits of nixtamalization conditions to modify the tortilla characteristics.

## Supplementary Information

Below is the link to the electronic supplementary material.

ESM1 (DOCX 0.99 MB)

## Data Availability

Data is available upon request.

## Abbreviations

BEβ: sheet structures
CO: Coils structures
CT: Cooking time
FTIR: Fourier transform infrared spectroscopy
IVPD: *In vitro* protein digestibility
LC: Lime concentration
PCA: Principal component analysis
PD: Protein digestibility
RDS: Rapidly digestible starch
RS: Resistant starch
SDS: Slowly digestible starch
TS: Total starch
T_x,y_: Tortilla treatments

## References

[1] Colín-Chávez C, Virgen-Ortiz JJ et al (2020) Comparison of nutritional properties and bioactive compounds between industrial and artisan fresh tortillas from maize landraces. Curr Res Food Sci 6(3):189–194. 10.1016/j.crfs.2020.05.004 10.1016/j.crfs.2020.05.004 PMC7473334 32914134

[2] Barquera S, Hernández-Barrera L, Trejo-Valdivia B et al (2020) Obesidad en México, prevalencia y tendencias en adultos. Ensanut 2018-19. Salud Pública 62(6):682–692. 10.21149/11630 10.21149/11630 33620965

[3] Svihus B, Hervik AK (2016) Digestion and metabolic fates of starch, and its relation to major nutrition-related health problems: a review. Starch-Stärke 68:302–313. 10.1002/star.201500295

[4] Aller EEJG, Abete I, Astrup A, Martinez JA, van Baak MA (2011) Starches, sugars and obesity. Nutrients 3(3):341–369. 10.3390/nu3030341 22254101 10.3390/nu3030341 PMC3257742

[5] Wang Y, Zhou X, Xiang X, Miao M (2022) Association of slowly digestible starch intake with reduction of postprandial glycemic response: an update meta-analysis. Foods 12(1):89. https://doi.org/10.3390%2Ffoods12010089 36613304 10.3390/foods12010089 PMC9818736

[6] Bojarczuk A, Skąpska S, Khaneghah AM, Marszałe K (2022) Health benefits of resistant starch: a review of the literature. J Funct Foods 93:105094. 10.1016/j.jff.2022.105094

[7] Rojas-Molina I, Nieves-Hernandez MG, Gutierrez-Cortez E et al (2024) Physicochemical changes in starch during the conversion of corn to tortilla in the traditional nixtamalization process associated with RS2. Food Chem 439:138088. 10.1016/j.foodchem.2023.138088 38064832 10.1016/j.foodchem.2023.138088

[8] van Soest JJ, Tournois H, de Wit D, Vliegenthart JF (1995) Short-range structure in (partially) crystalline potato starch determined with attenuated total reflectance Fourier-transform IR spectroscopy. Carbohydr Res 279:201–214. 10.1016/0008-6215(95)00270-7

[9] Walrafen GE, Hokmabadi MS, Yang WH (1986) Raman isosbestic points from liquid water. J Chem Phys 85:6964–6969. 10.1063/1.451383

[10] Méndez-Montealvo G, Trejo‐Espino JL, Paredes‐López O, Bello‐Pérez LA (2007) Physicochemical and morphological characteristics of nixtamalized maize starch. Starch‐Stärke 59(6):277–283. 10.1002/star.200600591

[11] Zhang W, He H, Tian Y, Gan Q, Zhang J, Yuan Y, Liu C (2015) Calcium ion-induced formation of β-sheet/-turn structure leading to alteration of osteogenic activity of bone morphogenetic protein-2. Sci Rep 5:12694. 10.1038/srep12694 26212061 10.1038/srep12694 PMC4515877

[12] Roldan-Cruz C, Garcia‐Diaz S, Garcia‐Hernandez A, Alvarez‐Ramirez J, Vernon‐Carter EJ (2020) Microstructural changes and *in vitro* digestibility of maize starch treated with different calcium compounds used in nixtamalization processes. Starch‐Stärke 72:1900303. 10.1002/star.201900303

[13] León-Villalobos JA, Maldonado‐Astudillo YI et al (2023) Effect of calcium hydroxide on pasting, thermal, and water‐adsorption behavior, and the flow properties of nixtamalized corn flour. J Food Process Eng 46:e14366. 10.1111/jfpe.14366

[14] Galán MG, Drago SR (2014) Effects of soy protein and calcium levels on mineral bioaccessibility and protein digestibility from enteral formulas. Plant Foods Hum Nutr 69:283–289. 10.1007/s11130-014-0432-y 25079612 10.1007/s11130-014-0432-y

[15] Baroroh U, Yusuf M, Rachman SD et al (2017) The importance of surface-binding site towards starch-adsorptivity level in α-amylase: A review on structural point of view. Enzyme Res 2017:4086845. 10.1155/2017/4086845 29359041 10.1155/2017/4086845 PMC5735674

[16] Vernon-Carter EJ, Meraz M, Bello-Perez LA, Alvarez-Ramirez J (2022) Analysis of starch digestograms using Monte Carlo simulations. Carbohydr Polym 291:119589. 10.1016/j.carbpol.2022.119589 35698344 10.1016/j.carbpol.2022.119589

[17] Acosta-Estrada BA, Serna-Saldívar SO, Chuck-Hernández C (2023) Nutritional assessment of nixtamalized maize tortillas produced from dry masa flour, landraces, and high yield hybrids and varieties. Front Nutr 10:1183935. 10.3389/fnut.2023.1183935 37485394 10.3389/fnut.2023.1183935 PMC10358733

[18] Dhital S, Shrestha AK, Gidley M (2010) Relationship between granule size and *in vitro* digestibility of maize and potato starches. Carbohydr Polym 82:480–488. 10.1016/j.carbpol.2010.05.018

[19] Sáyago-Ayerdi SG, Tovar J, Osorio-Diaz P, Paredes-López O, Bello-Pérez LA (2005) *In vitro* starch digestibility and predicted glycemic index of corn tortilla, black beans, and tortilla – bean mixture: effect of cold storage. J Agric Food Chem 53:1281–1285. 10.1021/jf048652k 15713053 10.1021/jf048652k

