# Supplementary material for: Effect of Calcium Hydroxide and Nixtamalization Time on the In Vitro Starch and Protein Digestibility of Traditional Maize Tortillas
Source: Plant Foods Hum Nutr. 2025 Feb 10;80(1):61. doi: 10.1007/s11130-024-01245-z (PMC11811476; doi:10.1007/s11130-024-01245-z)
Supplement: Supplementary file 1 — (DOCX 0.99 MB) [file 11130_2024_1245_MOESM1_ESM.docx]

**Supplementary Material**

**Effect of Calcium Hydroxide and Nixtamalization Time on the *in vitro* Starch and Protein Digestibility of Traditional Maize Tortillas**

I. Escamilla-Urbina,^1^ A. Totosaus-Sánchez (0000-0003-1965-9510),^1^ M.E. Rodríguez-Huezo (0000-0002-0638-6894)*^,1^, E.J. Vernon-Carter (0000-0001-8367-1499)^2^ and J. Alvarez-Ramirez (0000-0002-6482-889X)^2^

^1^Departamento de Ingeniería Química y Bioquímica, TecNM/TES Ecatepec, Av. Tecnológico S/N, Col. Valle de Anáhuac, Ecatepec de Morelos, Estado de México, C.P. 55210, MÉXICO.

^2^Departamento de Ingeniería de Procesos e Hidráulica. Universidad Autónoma Metropolitana-Iztapalapa. Apartado Postal 55-534, Iztapalapa, CDMX, 09340 México.

**Materials and Methods**

**Materials.** Maize (*Zea mays*, var. Celaya) grain was purveyed from a distributor in Ecatepec (State of Mexico, Mexico). It is a white maize native dent [1], containing about 25% amylose and 75% amylopectin, which is desired from grain varieties for tortilla production [2]. All reagents used were analytical grade and were purchased from J. T. Baker (Naucalpan, State of Mexico, Mexico). Enzymes for starch and protein digestion essays were obtained from Sigma-Aldrich Mexico (Toluca, State of Mexico, Mexico). All water used was distilled.

**Tortilla preparation.** Grain (1000 g) was mixed with 2 L of water added with either 1.0 or 2.0 g/100 g db calcium hydroxide. The mixture was cooked for 30, 45 or 60 min at boiling temperature (~ 92 ^o^C). The cooked grains were steeped for 12 h at room temperature. Then the cooking liquor was drained and the cooked maize grains were washed twice with water to remove the excess calcium and dissolved solids. The cooked maize grains were ground in a stone mill (Maquinova model MMQ06, Zapopan, State of Jalisco, Mexico), fitted with a screen (US Mesh 40, 0.4 mm) in the milling chamber, allowing appropriately coarse ground maize sized fractions to pass, retaining large fractions. Fresh masa was molded by pressure with a commercial press (Model MOTG, Tortilladoras Gonzalez, Naucalpan, Mexico) into thin circles to obtain 2 mm thick tortillas. Tortillas were cooked on a hot griddle for 1 min per side at 250±5 ^o^C [3]. The tortillas were coded as T_x,y._ where “x” denotes the lime concentration and “y” the maize grain cooking time used in the nixtamalization treatment.

**Fourier transform infrared spectroscopy.** The FTIR spectrum was obtained using an IR spectrophotometer (Cary 630, Agilent Technologies, Santa Clara, CA, USA) with an attenuated total reflectance (ATR) accessory. Dry samples were placed on the diamond crystal universal sample holder. The scan was performed in the range of 4000 to 400 cm^−1^ with a resolution of 4 cm^−1^. Numerical deconvolution with Gaussian basis functions (half-width of 15 cm^-1^, resolution enhancement 1.5) was carried to extract some features of the molecular organization of the main components of the maize tortillas.

**Total Starch**. Total starch (TS) was measured following AACC-2000 Method 76.13. To this end, 200 mg of tortilla milled samples were treated with 2 M KOH by 2 h to disperse all starch fractions. The sample was incubated with pancreatic α-amylase and amyloglucosidase for 16 h to hydrolyze the non-resistant starch. The reaction was stopped by the addition of ethanol. Finally, the samples were centrifuged to precipitate the non-hydrolyzed starch. The pellet was then treated with 2 M KOH and hydrolyzed by incubation with amyloglucosidase. The mixture was dried in an air oven (Double model OD302, Fisher & Paykel Appliances Ltd., Huntington Beach, CA, USA) at 45 °C for 24 h.

**In vitro digestibility.** *In vitro* multienzyme protein digestibility (IVPD) was determined as reported by [4]. Porcine pancreatic trypsin (Type IX, 15,310 units/mg protein), bovine b pancreatic chymotrypsin (Type II, 48 units/g solid), porcine intestinal peptidase (P-7500, 115 units/mg solid) and bacterial protease (Type XIV, 4.4 units/mg solid) were used for enzymatic digestion. Sample (~ 10 mg) was added to 10 mL of distilled water and ground with the help of a high shear homogenizer (Ultra-Turrax® T50 basic IKA Works, Inc., 164 Wilmington, DE, USA), operated at 6,000 rpm for 1.30 min, using an ice bath for avoiding temperature increase. Afterwards, the pH of the obtained mixture was adjusted to 8.0 with 1 N NaOH. One mL of enzyme aqueous solution (1.58 mg of trypsin, 3.65 mg of chymotrypsin and 0.45 mg of peptidase) was added to the protein sample and digestion was allowed to proceed for 10 min at 37 °C. After addition of 1 mL (1.48 mg) of bacterial protease solution, the digestion was continued for 9 min at 55 °C. The pH value was registered and used to estimate the IVPD according to the following expression:

$IVDP\left( \% \right)=234.84-22.56pH$ (1)

where $pH$ is the pH of the suspension [5].

*In vitro* starch digestion followed the methodology by Englyst et al. [6] with some modifications. Total starch (200 mg) isolated from tortilla milled samples were incubated with pancreatin from porcine pancreas (300 IU/mL, P1750) and amyloglucosidase (95 IU/mL, A7095) enzymes. A temperature of 37 °C and an incubation time of 120 min were used for both enzyme treatments. After hydrolysis for 20 or 120 min, the tubes were boiled for 10 min to deactivate the enzyme and terminate the reaction and centrifuged at 5000 ×g for 10 min. The glucose content in the supernatant was measured by the 3,5 dinitrosalicylic acid (DNS) method. The percentage of hydrolyzed starch was calculated by multiplying a factor of 0.9 to change values for glucose to starch. The percentage of the different hydrolyzed starch fractions were calculated with the following equations:

RDS (%) = (G_20_ × 0.9/W) × 100 (2)

SDS (%) = [(G_120_−G_20_) × 0.9/W] × 100 (3)

RS (%) = 100−RDS−SDS (4)

where G20 and G120 are glucose content released after 20 and 120 min, respectively; and W is the starch weight (mg) used for each test.

**Statistical Analyses**

All analyses were carried out in triplicate, and the results are expressed mean values with standard deviations. The experimental results were statistically analyzed by means of one-way ANOVA and Tukey's tests for statistical significance (p ≤ 0.05) with software SPSS Statistics 19.0.

**References**

1. Ramírez-Vega H, Vázquez-Carrillo G et al (2022) Physical and chemical characteristics of native maize from the Jalisco highlands and their influence on the nixtamalization process. Agriculture 12(9): 1293. https://doi.org/10.3390/agriculture12091293

2. Acosta-Estrada B A, Serna-Saldívar S O, Chuck-Hernández C (2023) Quality assessment of maize tortillas produced from landraces and high yield hybrids and varieties. Front Nutr 10: 1105619. https://doi.org/10.3389%2Ffnut.2023.1105619

3. Bello-Perez LA, Flores-Silva PC et al (2014) Effect of the nixtamalization with calcium carbonate on the indigestible carbohydrate content and starch digestibility of corn tortilla. J Cereal Sci 60(2): 421-425. https://doi.org/10.1016/j.jcs.2014.05.001

4. Elsohaimy S A, Refaay T M, Zaytoun M A M (2015) Physicochemical and functional properties of quinoa protein isolate. Ann Agric Sci 60: 297-305. <https://doi.org/10.1016/j.aoas.2015.10.007>

5. Martínez-Velasco A, Alvarez-Ramirez J et al (2018) Effect of the preparation method and storage time on the in vitro protein digestibility of maize tortillas. J Cereal Sci 84: 7-12. https://doi.org/10.1016/j.jcs.2018.09.016

6. Englyst H N, Kingman S M, Cummings J H (1992) Classiﬁcation and measurement of nutritionally important starch fractions. Eur J Clin Nutr 46: S33–S50. PMID 1330528

**Figure S.1**. FTIR spectra for the different tortilla formulations (T_x,y_) nixtamalized with: (a) x = 1.0 g/100 g db of calcium hydroxide and y = 30, 45, 60 min cooking time. (b) x = 2.0 g/100 g db of calcium hydroxide and 30, 45, 60 min cooking time.

**Figure S.2**. Illustration of the numerical deconvolution of the (a) OH, (b) Amide I and (c) starch FTIR bands.

**Figure S.3**. Protein hydrolysis kinetics of the tortilla formulations (T_x,y_) nixtamalized with: (a) x = 1.0 g/100 g db of calcium hydroxide and y = 30, 45, 60 min cooking time. (b) x = 2.0 g/100 g db of calcium hydroxide and 30, 45, 60 min cooking time.The lines denote piecewise least-square linear fittings. The lines denote the least-squares fitting by either linear or exponential functions.

**Figure S.4**. Digestograms of the tortilla formulations (T_x,y_) nixtamalized with: (a) x = 1.0 g/100 g db of calcium hydroxide and y = 30, 45, 60 min cooking time. (b) x = 2.0 g/100 g db of calcium hydroxide and 30, 45, 60 min cooking time. The lines denote piecewise least-square linear fittings.
